# A Comparison Between Two Assays for Measuring Seminal Oxidative Stress and their Relationship with Sperm DNA Fragmentation and Semen Parameters

**DOI:** 10.3390/genes10030236

**Published:** 2019-03-19

**Authors:** Sheryl T. Homa, Anna M. Vassiliou, Jesse Stone, Aideen P. Killeen, Andrew Dawkins, Jingyi Xie, Farley Gould, Jonathan W. A. Ramsay

**Affiliations:** 1 Department of Biosciences, University of Kent, Canterbury CT2 7NJ, UK; anna@andrologysolutions.co.uk (A.M.V.); jlstone19@gmail.com (J.S.); 2 Department of Andrology, The Doctors Laboratory, London W1G 9RT, UK; aideen.killeen@tdlpathology.com (A.P.K.); andrew.dawkins@tdlpathology.com (A.D.); jingyi-xie@hotmail.co.uk (J.X.); FGould@hotmail.co.uk (F.G.); 3 Imperial College Healthcare NHS Trust, London W2 1NY, UK; jonathan.ramsay@imperial.nhs.uk

**Keywords:** oxidative stress, reactive oxygen species, chromatin, DNA fragmentation, DNA oxidation, male infertility, spermatogenesis

## Abstract

Oxidative stress (OS) is a significant cause of DNA fragmentation and is associated with poor embryo development and recurrent miscarriage. The aim of this study was to compare two different methods for assessing seminal OS and their ability to predict sperm DNA fragmentation and abnormal semen parameters. Semen samples were collected from 520 men attending for routine diagnostic testing following informed consent. Oxidative stress was assessed using either a chemiluminescence assay to measure reactive oxygen species (ROS) or an electrochemical assay to measure oxidation reduction potential (sORP). Sperm DNA fragmentation (DFI) and sperm with immature chromatin (HDS) were assessed using sperm chromatin structure assay (SCSA). Semen analysis was performed according to WHO 2010 guidelines. Reactive oxygen species sORP and DFI are negatively correlated with sperm motility (*p* = 0.0012, 0.0002, <0.0001 respectively) and vitality (*p* < 0.0001, 0.019, <0.0001 respectively). The correlation was stronger for sORP than ROS. Reactive oxygen species (*p* < 0.0001), sORP (*p* < 0.0001), DFI (*p* < 0.0089) and HDS (*p* < 0.0001) were significantly elevated in samples with abnormal semen parameters, compared to those with normal parameters. Samples with polymorphonuclear leukocytes (PMN) have excessive ROS levels compared to those without (*p* < 0.0001), but sORP and DFI in this group are not significantly increased. DNA fragmentation was significantly elevated in samples with OS measured by ROS (*p* = 0.0052) or sORP (*p* = 0.004). The results demonstrate the multi-dimensional nature of oxidative stress and that neither assay can be used alone in the diagnosis of OS, especially in cases of leukocytospermia.

## 1. Introduction

Oxidative stress (OS) is thought to be the pathologic molecular mechanism underpinning the majority of known clinical, environmental and lifestyle causes of male infertility. It is associated with varicocoele, genitourinary tract infection, prostatitis, obesity, tobacco smoking, endocrine imbalance and testicular dysfunction [1,2,3,4]. OS occurs when the physiological balance of oxidants and reductants in a system is disturbed as a result of excessive production of reactive oxygen species (ROS) or a reduction in levels of antioxidants [5]. Oxidative balance is essential for normal sperm function [2,4,6,7,8,9]. Reactive oxygen species include superoxide anion (O_2_^−^^•^), hydroxyl radical (OH•), hydrogen peroxide (H_2_O_2_), peroxynitrite (ONOO−), nitric oxide (•NO) or hypochlorous acid (HOCl). Reactive oxygen species are required at low levels for chromatin and flagellar protein modification during spermatogenesis [10] as well as for the normal process of sperm hyperactivation and capacitation [2,6,7,8,9]. However, at high levels, OS impairs fertilisation through interference with capacitation and the acrosome reaction [4,6,8].

Sperm are exposed to OS during spermatogenesis as well as during epididymal storage and transit through the reproductive tract and at ejaculation [4,8,11]. Reactive oxygen species may be produced extrinsically by infiltrating polymorphonuclear leukocytes (PMN) [12,13,14,15] or from the presence of oxidants in the seminal fluid resulting from tobacco smoking, excessive testicular heat or other environmental toxins [3,4]. On the other hand, ROS can be generated intrinsically, primarily as a result of electron leakage in the sperm mitochondria, from cytosolic L-amino acid oxidases and plasma membrane nicotinamide adenine dinucleotide phosphate (NADP) oxidases [6,16,17]. High levels of ROS are also produced by abnormal sperm that retain excess residual cytoplasm as a result of incomplete sperm maturation [6,18]. As the sperm have negligible sources of intracellular antioxidants, ROS levels may remain elevated, leaving vulnerable molecules susceptible to oxidative attack [6,8]. Reactive oxygen species are extremely harmful because they target every cellular constituent, which has serious consequences for cell signalling and the function of the sperm. The sperm plasma membrane is particularly susceptible to oxidation as it is enriched in polyunsaturated fatty acids. These lipids can be oxidised through a series of chain reactions to release potentially toxic and mutagenic aldehydes and alkenals [6,15,19,20]. Importantly, sperm DNA is exquisitely sensitive to oxidative attack, resulting in impairment of embryo development, increased risk of gene mutations and miscarriage, congenital malformations and a high frequency of diseases in the offspring [3,5,8,11,19,21,22]. A serious consequence of OS is that it interferes with epigenetic modification and there are reports of abnormalities in sperm gene methylation as a direct result of oxidative insult [23,24,25]. There is good evidence to link unexplained infertility and recurrent pregnancy loss with both oxidative stress and sperm DNA fragmentation which are significantly elevated in infertile men [26,27,28,29] and in men whose partners experience miscarriage [11,30,31].

For many decades, semen analysis has been considered the gold standard for assessment of male infertility. However, this subjective microscopic analysis is poorly correlated with infertility and fails to provide any information about sperm function. More recently, assessment of sperm DNA fragmentation has been implemented as a more reliable marker for male infertility [32], yet it also has limitations as it does not address the plethora of other physiological and pathological functions regulated by oxidative stress in sperm. Assessment of OS can be performed in semen using a chemiluminescence assay [33,34,35]. This test measures the oxidation of luminol, a chemiluminescent probe, providing information about the levels of oxidants in the system. Alternatively, oxidative stress can be measured using a novel electrochemical assay which determines the oxidation reduction potential of the system taking into consideration all of the oxidants and antioxidants that are present [36,37]. The latter test is a simpler and more efficient method to determine OS. However, the two tests look at different aspects of oxidative stress, but it is precisely because of this that we consider it important to determine which assay may be more clinically relevant if we are to implement them as useful diagnostic tests. In order for an assay to be accepted as a clinically relevant diagnostic test, it should be able to predict abnormalities in markers known to be affected by it. While previous studies of the chemiluminescence and redox assays have shown an association of OS with clinical markers such as semen parameters [28,38,39,40,41] and sperm DNA fragmentation [40,42,43,44,45,46], to date a comparison of the two assays has not been performed. This study presents the first comparison of the two assays to determine which assay is more predictive of impaired semen quality. The association of oxidative stress markers (ROS and sORP) with sperm DNA fragmentation and semen parameters was investigated. Given that leukocytes are a major source of extrinsic ROS and that the role of leukocytes in male infertility remains controversial, the assays were also compared between samples with and without leukocytospermia. The results show a clear association between OS and sperm DNA damage, as well as impaired semen parameters irrespective of the method used to measure OS, although sORP is more predictive than ROS, especially in cases of leukocytospermia. It is proposed that differences between the methods may be explained in part by a difference in sensitivity for measurement of OS in extrinsic versus intrinsic compartments of the sperm in seminal fluid.

## 2. Materials and Methods

### 2.1. Ethics Statement

This study was approved by the Faculty of Sciences Research Ethics Advisory Group for Human Participants at the University of Kent (ID number 0601516) and adhered to the current legislation on research involving human subjects in the UK.

### 2.2. Semen Samples

Semen samples were collected from men who were attending for diagnostic semen analysis and who had given their informed consent to use any of the sample that remained after analysis for the study. Participants were advised to have 2–5 days sexual abstinence before providing a sample on site. A total of 599 samples were provided for the study, of which 79 were excluded. Exclusion criteria were incomplete sample collection, febrile illness during the previous 12 weeks, both of which may have affected the reliability of the results, and samples containing less than 1 million/mL sperm as ROS and sORP measurement are inaccurate and unreliable when the sperm concentration falls below this value. Of the men who consented, 496 were included in the study. Of these, 24 had attended for a repeat semen analysis following a clinical management plan. Semen analysis was performed as part of diagnostic testing according to the WHO 2010 criteria [47]. Samples were incubated at 36°C (±1 °C) to liquefy prior to analysis. All samples were analysed at 20 ± 5 min after production. Leukocytes were identified using a peroxidase screen (LeucoScreen, FertiPro N.V. Belgium) and differential cell counting on Papanicoloau stained slides assessed under oil immersion at ×1000 magnification. The distribution of semen samples in the study cohorts is shown in Table 1. The heterogeneity of the semen samples is evenly distributed among the OS study groups. Teratozoospermia was the most prevalent abnormality in both study groups, constituting 24% of all samples. Less than 10% of samples assessed for OS had abnormalities in all three major semen parameters. 

### 2.3. MeasuringReactive Oxygen Species Using Chemiluminescence

Reactive oxygen specieslevels were measured using a CE-marked single-tube Luminometer (Modulus Model no. 9200-001; Turner Biosystems Instrument Inc., Sunnyvale, CA, USA). Luminol was used as the probe, which is oxidised in the presence of ROS, resulting in chemiluminescence. The general methodology for this test has been reviewed elsewhere [34,35]. Briefly, negative and positive controls were run daily. Negative controls were prepared using 400 μL phosphate buffered saline (PBS) with 10 μL of a luminol working solution (Sigma-Aldrich, Dorset, UK) (5 mM luminol prepared in dimethylsulphoxide (DMSO)). Positive controls were prepared using 395 μL PBS, 5 μL 30% H_2_O_2_ and 10 μL of 5 mM luminol working solution. When measuring ROS in semen, 10 μL of luminol working solution was added to 400 μL of liquefied whole semen at 20 min post-ejaculation and measured immediately. Results were normalized to the sperm concentration and reported in relative light units (RLU)/sec/10^6^ sperm.

### 2.4. Measuring Oxidation-Reduction Potential Using MiOXSYS

The ORP of semen samples was measured using the CE-marked MiOXSYS platform (MiOXSYS, Aytu BioScience Inc., Englewood, CO, USA). The analyser consists of an ultrahigh impedance electrometer with a self-contained electrochemical cell with platinum working and counter electrodes, and a 3 M KCl, Ag/AgCl reference electrode [48]. For the assay, a 30 µL sample is applied to a sensor which is inserted into the analyser. The voltage is measured between the reference and working electrodes every 0.5 s. The final sORP (static oxidation reduction potential) reading on the analyser display screen is the average of the final 10 s (20 readings). Higher sORP readings indicate an imbalance that favours the pro-oxidants and therefore suggests the presence of oxidative stress in the sample [48]. A reading in mV is displayed on the MiOXSYS analyser. This value is normalised to the sperm concentration of the sample. The result is reported as mV/10^6^ sperm/mL.

### 2.5. Measuring Sperm DNA Fragmentation

The sperm chromatin structure assay (SCSA^®^) was utilized to assess DNA fragmentation. Details of the SCSA^®^ have been described in detail elsewhere [32]. In brief, sperm are treated with a low pH buffer for 30 sec that opens up the two DNA strands where there is either a single (sd) or double (ds) DNA strand break. Acridine orange complexes with ds DNA and fluoresces green while complexing with sd DNA produces red fluorescence. Those sperm with any measurable increase in red fluorescence are scored as sperm with DNA fragmentation (DFI). Additionally, we included assessment of the fraction of high DNA stainable (HDS) cells, which are considered to represent immature spermatozoa with incomplete chromatin condensation.

### 2.6. Statistical Analysis

All data were analysed using the Statistical Analysis Systems software package (SAS Inst. Inc., Cary, NC) version 9.4. Data were tested for adherence to normality using PROC UNIVARIATE (SAS, 2013). The CORR procedure of SAS (PROC CORR, SAS 2013) was used to determine correlations between various semen parameters. Pearson correlation coefficients (R^2^) and *p* values were estimated and reported for all parameters. Due to the expected non-normality of quantitative variables in this study, group comparisons were performed with Kruskal–Wallis test for 3-group comparisons, or Wilcoxon rank sum test for pairwise group comparisons using the NPAR1WAY procedure of SAS (PROC NPAR1WAY, SAS 2013). These nonparametric tests were used for age, sperm concentration/mL, total motility, progressive motility, vitality, morphology, PMN concentration, ROS, sORP, DFI and HDS values. In all cases, *p* values < 0.05 were considered to be statistically significant.

## 3. Results

### 3.1. Correlation between OS and Sperm DNA Damage; Comparison between Two Methods of OS Measurement

This study investigated whether there is a direct correlation between OS and DNA damage and whether the observations are consistent between the two methods of OS measurement. Observations were made with and without inclusion of samples with leukocytospermia as they are known to generate high levels of exogenous ROS and may obscure the effects of ROS generated endogenously. Initially, it was necessary to determine whether detection of OS was comparable between the two methods of assessment. Oxidative stress was assessed in 315 samples using either the chemiluminescence assay or oxidation reduction potential assay. Results showed only a weak but nevertheless significant positive correlation between observations for ROS and sORP (R^2^ = 0.1172, *p* = 0.0376, n = 315). Interestingly, when samples with leukocytospermia are excluded, the correlation between sORP and ROS is marginally stronger (R^2^ = 0.15095, *p* = 0.0089, n = 299). When OS was compared to DFI levels, ROS was highly significantly correlated to DFI, exhibiting a moderate positive relationship (R^2^ = 0.24316, *p* = 0.0002, n = 237). Oxidation reduction potential shows a similar relationship to DFI, however this is not significant and may be due to the relatively low sample numbers (R^2^ = 0.23992, *p* = 0.1043, n = 47). The ROS versus DFI correlation is also slightly stronger in the absence of data from patients exhibiting elevated PMN (R^2^ = 0.31139, *p* < 0.0001, n = 222), however this is not the case for sORP. This is likely because only one patient from the sORP group exhibited leukocytospermia (R^2^ = 0.22706, *p* = 0.1291, n = 46). In contrast, HDS shows no significant correlation with oxidative stress, whether it is measured by ROS (R^2^ = 0.11211, *p* = 0.085, n = 237) or sORP (R^2^ = 0.01222 *p* = 0.9351, n = 47), irrespective of leukocytospermia (excluding PMN: ROS R^2^ = 0.10329, *p* = 0.1249, n = 222; sORP R^2^ = 0.01853, *p* = 0.9027, n = 46).

### 3.2. Sperm DNA Damage and HDS Levels in Oxidative Balanced versus Oxidative Stressed Semen Samples

The chemiluminescence and MiOXSYS assays have both been validated and verified in-house at The Doctors Laboratory, which is ISO15189 UKAS accredited. The reference ranges determined by ROC analysis were ≤13.8 RLU/sec/10^6^ sperm/mL (86% sensitivity; 86% specificity) for ROS and ≤1.4 mV/10^6^ sperm/mL (76.4% sensitivity; 75.9% specificity) for sORP. Samples are considered to be in oxidative stress if they exceed the clinical reference values. When the patient cohort is separated into groups with or without oxidative stress, mean DFI (Table 2) was significantly elevated in the OS group irrespective of the method of OS measurement used, although the difference was much more significant when OS was measured by redox potential. When samples with PMN are excluded, the DFI is slightly higher in the group with OS as measured by ROS (24.67 ± 1.78 vs. 22.86 ± 1.59), but this difference was not significant. There were no samples with leukocytospermia in the group with OS as measured by sORP. While %HDS was also significantly elevated in samples with high levels of ROS (Table 3), the increase seen in %HDS in samples with elevated sORP was not significant, which may be attributed to the lower numbers of samples in this test group.

### 3.3. Correlation between Oxidative Stress, Sperm DNA Damage and Semen Parameters

Oxidative stress is manifested in poor semen quality. Using the two different methods for measuring OS, the results demonstrate a highly significant negative correlation between OS and total motility, progressive motility, total motile sperm count, vitality and morphology (see Table 4). The correlation is approximately twice as strong when OS is measured by sORP compared to ROS for all parameters with the exception of vitality which shows a stronger and more significant correlation with ROS (sORP: R^2^ = −0.13519, *p =* 0.019; ROS: R^2^ = −0.20832, *p* < 0.0001). This indicates that measurement of sORP may be a more sensitive marker for oxidative stress than ROS. An even stronger, highly significant negative correlation is seen between DFI and semen parameters, particularly with total (R^2^ = −0.53951, *p* = <0.0001) and progressive motility (R^2^ = −0.48693, *p* < 0.0001) and vitality (R^2^ = −0.5727, *p* < 0.0001).

In contrast, HDS levels are not correlated with vitality, but are negatively correlated with all other semen parameters. The strongest correlation is between HDS and morphology and is highly significant (R^2^ = −0.48848, *p =* 0 < 0.0001). Oxidation reduction potential is also highly significantly negatively correlated to morphology (R^2^ = −0.22642, *p* = <0.0001), although not as strong a correlation as between HDS and morphology. Polymorphonuclear leukocytes are known to produce high levels of ROS, however presence of PMN in seminal fluid shows no correlation with classical markers of oxidative damage in sperm, including motility, vitality and DNA damage (see Table 4), although there is a strong positive correlation with sperm count.

### 3.4. Comparison of Sperm DNA Damage and OS among Different Patient Groups Selected According to Semen Parameters

**Figure 1:**
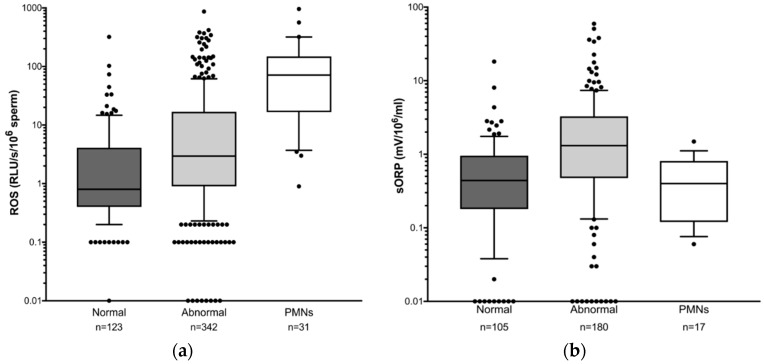
Box and whisker plots for seminal oxidative stress levels in different patient groups showing the median and interquartile ranges. Normal—normal semen parameters; Abnormal—abnormal parameters with <1 million/mL leukocytes; PMN—any parameters with ≥1 million/mL leukocytes. OS was measured using either (**a**) chemiluminescence (ROS) or (**b**) oxidation reduction potential (sORP) Lower whisker = 10th percentile; upper whisker = 90th percentile. Dots indicate values outside the range. Data are shown on a logarithmic scale.

To further evaluate the correlation between OS, DNA damage and semen parameters, patients were grouped according to whether they had normal or abnormal semen parameters. As PMN are well known to generate high levels of ROS, samples containing ≥1 × 10^6^ million/ml PMN were grouped in a separate category. Reactive oxygen species, sORP, DFI and HDS levels were analysed between the different patient groups. Figure 1 shows OS levels are significantly higher in semen samples with one or more abnormal semen parameters compared to samples with normal semen parameters as expected, irrespective of the method of OS measurement (Figure 1a ROS: *p* < 0.001; Fig 1b sORP: *p* < 0.007). Median ROS levels are 0.80 (range 0–319.6) for normal semen samples versus 2.95 (range 0–1755) for abnormal samples, while median sORP is 0.44 (range −0.18–18.16) for normal samples versus 1.31 (range −0.78–59.43) for abnormal samples.

Reactive oxygen species levels are highest in the group of men with leukocytospermia and are significantly higher than in men with normal semen parameters (PMN: median 71.3, range 0.9–957.2 vs. normal: 0.8, range 0–319.6) (*p* < 0.0001). Unexpectedly, unlike the results observed with ROS measurement, sORP levels are not significantly different between the group with normal semen parameters and the group with PMN (Figure 1b sORP normal: median 0.44, range −0.18–18.16 vs. PMN: median 0.40, range 0.06–1.49).

**Figure 2:**
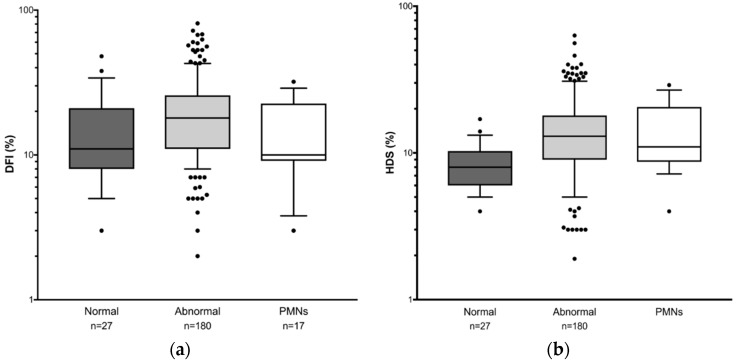
Box and whisker plots for sperm genetic integrity in different patient groups showing the median and interquartile ranges. Normal—normal semen parameters; Abnormal—abnormal parameters with <1 million/mL leukocytes; PMN—any parameters with ≥1 million/mL leukocytes. Sperm DNA fragmentation and HDS were assessed in the same samples. (**a**) DNA fragmentation (DFI) (**b**) immature chromatin (HDS). Lower whisker = 10th percentile; upper whisker = 90th percentile. Dots indicate values outside the range. Data are shown on a logarithmic scale.

Figure 2 shows the difference in sperm DNA damage between patient groups. While median DFI is significantly higher in samples with abnormal semen parameters compared to those with normal parameters (Figure 2a: median 18; range 2–81 vs. median 11; range 3–48 respectively; *p* = 0.0089), as with sORP, median DFI in the leukocytospermia samples (10; range 3–32) is not significantly different to those with normal semen parameters.

Interestingly, the mean age of men in the PMN group is significantly higher than in the other two groups (PMN 41.50 ± 1.34 vs. normal 38.46 ± 0.57, *p* = 0.0342; vs. abnormal 38.25 ± 0.34, *p* = 0.0134). As it has previously been reported that sperm DNA becomes increasingly fragmented as men age [3,49], one might expect that samples in the leukocytospermia group would exhibit the highest levels of sperm DNA damage, but clearly this is not the case. On the other hand, HDS levels are significantly raised in both the leukocytospermic and abnormal semen parameter groups compared to samples with normal semen parameters (Figure 2b; normal: median 8; range 4–17 vs. abnormal: median 13; range 1.9–63.1 *p* = < 0.0001; vs. PMN: median 11; range 4–29 *p* = 0.0075) with no overlap between the boxes, suggesting a different mechanism of action for sperm DNA damage and sperm genetic maturation.

## 4. Discussion

A systematic review of the literature comparing different methods for measuring OS in terms of the practicality of the methodology of the tests in the laboratory, cost effectiveness and sensitivity and specificity of the tests has revealed the superiority of the MiOXSYS assay [50]. Our study expands on the review by Agarwal et al. [50] as it is the first to perform a comparison of two different tests for measuring oxidative stress (chemiluminescence versus redox potential) in terms of their efficacy in predicting impaired semen parameters and sperm chromatin structure, and hence their usefulness as a clinical diagnostic test. Novel findings from our study demonstrate that redox potential is more highly correlated with semen parameters than measurement of ROS. Furthermore, established clinical reference ranges for OS tests are extremely useful in categorising patients with altered sperm chromatin structure or sperm DNA damage, when the DFI threshold is 25%. We have shown for the first time that this is particularly more evident for those patients exhibiting OS as measured by redox potential. This study also investigated the ambiguity surrounding the measurement of oxidative stress in leukocytospermia. We compared oxidative stress measured by chemiluminescence versus oxidation reduction potential in the presence and absence of leukocytospermia. The data we report for the association between leukocytes and the MiOXSYS assay is entirely novel. We did not observe any difference in levels of sORP or DNA fragmentation in samples with or without leukocytospermia, which was unexpected, especially as leukocytes are a significant source of ROS.

The correlation between the two methods for measuring OS is not strong and this is not surprising since the assays measure different aspects of OS. Chemiluminescence measurement of OS specifically detects ROS [51,52]. In contrast, measurement of redox potential generates a single measurement from the culmination of the plethora of oxidation-reduction reactions occurring within a biological system [53]. Given that the two methods assess OS from different perspectives, it is important to establish that they are both relevant biomarkers of sperm pathology in order for them to be useful as diagnostic tests for infertility.

There is a moderate correlation between ROS and DFI and it is highly significant, while the correlation between sORP and DFI is not significant. Only one previous study has examined the relationship between sORP and DNA fragmentation [45], but DNA damage was measured by sperm chromatin dispersion rather than SCSA as in this study. They showed that while sORP and sperm DFI were not correlated in fertile men, there was a significant correlation in infertile men [45]. Since the fertility status of the men in this study was unknown, it is possible that the samples assessed for sORP contained a higher proportion of men who were fertile. The relationship between OS and DFI becomes considerably more apparent when samples are grouped according to whether they are considered to be in oxidative balance or OS. Under these circumstances, there is a significant increase in sperm DNA damage in semen samples that exhibit OS, whether OS is measured by ROS or sORP, although this is particularly significant when OS is measured by sORP. There is a wealth of evidence demonstrating an association between sperm DNA damage and OS assessed by chemiluminescence [40,42,43,44,52], but this is the first study to report an increase in sperm DNA damage in samples exposed to OS as determined by the MiOXSYS assay.

Oxidative stress is well known to manifest its effects on semen parameters [28,38,39,40,41,46], as well as playing a major role in sperm DNA damage [8,21,22,27,30,40,42,43,44,54]. In this study, while we have shown that both sORP and ROS are significantly negatively correlated with semen parameters, the correlation is approximately twice as strong for all parameters, with the exception of vitality, when OS is measured by sORP. Sperm DNA fragmentation is also significantly negatively correlated with all semen parameters, particularly with motility and vitality, in agreement with previous findings [49,55,56]. Interestingly, sperm DNA fragmentation shows a much stronger negative correlation than OS with sperm motility and vitality, indicating an alternative source for DNA damage that does not involve OS. Indeed, high DFI levels in some samples are not accompanied by elevations in sORP or ROS. Although sperm DNA damage and subsequent loss of vitality is a major consequence of OS [54], oxidative damage is only one of several etiologies that are responsible for DNA fragmentation, including abnormal caspase activity leading to abortive apoptosis, incomplete protamination and chromatin packaging abnormalities and anomalies in endonuclease and topoisomerase II activity [6,8,11].

The association of OS and sperm DNA damage with semen parameters is further highlighted in this study by the higher levels of these biomarkers seen in semen from patients with abnormal semen parameters compared to those with normal semen parameters. A significant reduction in semen parameters and a significant increase in seminal ROS have previously been demonstrated in infertile men [28]. In addition, sORP was elevated in men with abnormal semen parameters [57], and a significant correlation between morphology and sORP has been demonstrated in infertile men [45]. We have shown that samples with abnormal semen parameters are more likely to have elevated sORP and ROS, with increased DFI and HDS. However, the relationship between sORP, ROS and DNA damage is more complex and may be dependent upon the source of oxidants. The results presented in this study corroborate previous findings demonstrating excessive production of ROS by PMN [12,14,15,33,41,58], but on the contrary, sORP is not elevated in samples with leukocytospermia, and there is no evidence for any significant increase in sperm DNA fragmentation. On the other hand, measurement of sperm DNA fragmentation is not a true measurement of DNA oxidation as it assesses DNA strand breaks only, and oxidation of sperm DNA with ensuing pathology may occur in the absence of any measurable levels of sperm DNA fragmentation. Furthermore, the presence of leukocytes does not appear to cause any damage to sperm parameters in this study. The role of leukocytes in male infertility remains controversial. Some studies reveal no correlation between seminal PMN and semen parameters [59] or sperm DNA damage [14,58,59] assessed by either TUNEL or 8--OHDG, while two studies demonstrated a significant positive correlation between PMN and DNA damage [12,60] assessed by TUNEL and SCSA respectively, and two studies showed negative correlations between PMN and semen parameters [12,58]. One explanation for this could be that there is a temporal lag between release of ROS and the time at which physiological effects in sperm are manifested. Since the origin of seminal leukocytes is primarily from the male accessory glands [61], exposure of spermatozoa to ROS produced by leukocytes would only occur at ejaculation, with insufficient time to have any significant effect on semen quality. However, this does not explain the low levels of sORP in samples with leukocytospermia, even though PMN are producing exceptionally high levels of ROS.

Alternatively, it could be argued that the detrimental effects of OS on sperm are due to the location of ROS production. ROS are produced exogenously by PMN within the seminal fluid milieu, which is normally enriched in antioxidants [62]. Hence the effects of the oxidants are mitigated before they come into contact with the sperm. Alternatively, OS produced intrinsically by sperm themselves are more likely to cause DNA damage as spermatozoa contain negligible antioxidants making the internal components highly susceptible to oxidative damage [19]. Taken together, these observations may serve to explain why seminal ROS levels are high and sORP levels are relatively low in the presence of PMN, since PMN are a source of exogenous ROS, and sORP measurement takes into account the levels of antioxidants as well as pro-oxidants in the system. It may also explain why the correlation between OS and semen parameters is much stronger when OS is measured by sORP, and why the increase in DNA damage in the group with OS is more significant when OS is measured using sORP.

Another marker of sperm genetic integrity is that of high DNA stainability (HDS), which measures sperm with abnormal protamination of chromatin where nuclear histones are retained. We observed a strong negative correlation between HDS and sperm morphology confirming previous observations that sperm head abnormalities can be caused by protamine deficiency, incomplete protamine sulfhydryl oxidation and chromatin condensation [63,64]. The origin of aberrant protamination in the sperm nucleus is unclear. Although we did not observe any correlation between HDS and OS, HDS levels were increased in samples with OS compared to those that did not exhibit OS, but the difference is only significant when OS is measured by ROS. Furthermore, unlike DFI, HDS is significantly higher in leukocytospermia consistent with previous studies confirming a role for extrinsic ROS in sperm nuclear chromatin compaction [65,66].

Overall, the stronger association of sORP with both DNA fragmentation and semen parameters lends support to the view that measurement of redox potential is a more powerful tool than chemiluminescence for determining the pathological oxidative state of the sperm. The severity of the pathological consequences of oxidative stress on sperm highlights the importance of measuring OS as the most influential marker of sperm function. However, more work is warranted to establish the proposed theoretical model of extrinsic and intrinsic ROS production and whether leukocytospermia contributes to sperm DNA fragmentation.

## Figures and Tables

**Table 1:** Distribution of semen samples classified according to WHO (2010) criteria in the patient study cohorts.

	ROS		sORP	
	Number of Patients	%	Number of Patients	%
Normozoopsermia	172	34.6	139	46.2
Oligozoospermia	18	3.6	9	3.0
Asthenozoospermia	8	1.6	6	2.0
Teratozoospermia	119	24.0	72	23.9
Oligoasthenozoospermia	1	<1	1	<1
Oligoteratozoospermia	69	13.9	32	10.6
Asthenoteratozoospermia	29	5.8	11	3.7
Oligoasthenoteratozoospermia	49	9.9	15	5.0
Leukocytospermia	31	6.3	16	5.3
TOTAL	496	-	301	-

ROS = reactive oxygen species; sORP = static oxidation reduction potential.

**Table 2:** Sperm DNA fragmentation in the presence and absence of oxidative stress.

	All Samples	All Samples Excluding those with Leukocytospermia
**Oxidative stress measured by ROS**		
Oxidative balanced	18.78 ± 1.10 (161)	18.75 ± 1.12 (159)
Oxidative stressed	22.86 ± 1.59 (77)	24.67 ± 1.78 (63)
*p* value	0.0359	0.0052
**Oxidative stress measured by sORP**		
Oxidative balanced	11.97 ± 1.41 (30)	12.14 ± 1.49 (28)
Oxidative stressed	19.39 ± 1.83 (18)	19.39 ± 1.86 (18)
*p* value	0.0024	0.004

Values represent the mean %DFI ± SEM. Number of samples in parentheses.

**Table 3:** High DNA stainability of sperm in the presence and absence of oxidative stress.

	All Samples	All Samples Excluding those with Leukocytospermia
**Oxidative stress measured by ROS**		
Oxidative balanced	13.45 ± 0.74 (161)	13.49 ± 0.75 (159)
Oxidative stressed	15.78 ± 1.02 (77)	16.19 ± 1.16 (63)
*p* value	0.0097	0.0077
**Oxidative stress measured by sORP**		
Oxidative balanced	11.07 ± 1.11 (30)	10.61 ± 1.09 (28)
Oxidative stressed	17.89 ± 3.40 (18)	17.89 ± 3.40 (18)
I value	0.0881	0.0672

Values represent the mean %HDS ± SEM. Number of samples in parentheses.

**Table 4:** Correlation between oxidative stress, sperm genetic integrity and semen parameters.

	Value	Count/ml	Total Motility	Progressive Motility	Total Motile Sperm Count	Vitality	Morphology
ROS	R^2^	−0.15729	−0.14482	−0.14444	−0.17395	−0.20832	−0.12536
	*p* value	0.0004	0.0012	0.0013	0.0001	<0.0001	0.0053
	n	496	495	495	495	495	493
sORP	R^2^	−0.24628	−0.21101	−0.23561	−0.25055	−0.13519	−0.22642
	*p* value	<0.0001	0.0002	<0.0001	<0.0001	0.019	<0.0001
	n	301	301	301	301	301	300
DFI	R^2^	-0.19182	−0.53951	−0.48693	−0.27539	−0.5727	−0.19016
	*p* value	0.0041	<0.0001	<0.0001	<0.0001	<0.0001	0.0047
	n	222	221	221	221	221	220
HDS	R^2^	−0.36663	−0.23638	−0.24938	−0.27703	−0.11497	−0.48848
	*p* value	<0.0001	0.0004	0.0002	<0.0001	0.0882	<0.0001
	n	222	221	221	221	221	220
PMN	R^2^	0.2098	0.03497	0.04169	0.15498	0.03037	0.04361
	*p* value	<0.0001	0.4389	0.3561	0.0006	0.5015	0.3354
	n	493	492	492	492	492	490

R^2^ = Pearson correlation coefficients; n = number of samples. ROS = Reactive oxygen species; sORP = oxidation-reduction potential; DFI = DNA fragmentation index; HDS = sperm with immature chromatin; PMN = polymorphonuclear leukocytes.
